# Computational Mechanisms Underlying Motivation to Earn Symbolic Reinforcers

**DOI:** 10.1523/JNEUROSCI.1873-23.2024

**Published:** 2024-04-26

**Authors:** Diana C. Burk, Craig Taswell, Hua Tang, Bruno B. Averbeck

**Affiliations:** Laboratory of Neuropsychology, National Institute of Mental Health, National Institutes of Health, Bethesda, Maryland 20892-4415

**Keywords:** computational modeling, learning, Markov decision process, motivation, reinforcement learning, reward

## Abstract

Reinforcement learning is a theoretical framework that describes how agents learn to select options that maximize rewards and minimize punishments over time. We often make choices, however, to obtain symbolic reinforcers (e.g., money, points) that are later exchanged for primary reinforcers (e.g., food, drink). Although symbolic reinforcers are ubiquitous in our daily lives, widely used in laboratory tasks because they can be motivating, mechanisms by which they become motivating are less understood. In the present study, we examined how monkeys learn to make choices that maximize fluid rewards through reinforcement with tokens. The question addressed here is how the value of a state, which is a function of multiple task features (e.g., the current number of accumulated tokens, choice options, task epoch, trials since the last delivery of primary reinforcer, etc.), drives value and affects motivation. We constructed a Markov decision process model that computes the value of task states given task features to then correlate with the motivational state of the animal. Fixation times, choice reaction times, and abort frequency were all significantly related to values of task states during the tokens task (*n* = 5 monkeys, three males and two females). Furthermore, the model makes predictions for how neural responses could change on a moment-by-moment basis relative to changes in the state value. Together, this task and model allow us to capture learning and behavior related to symbolic reinforcement.

## Significance Statement

Symbolic reinforcers, like money and points, play a critical role in our lives. However, we lack a mechanistic understanding of how symbolic reinforcement can be related to fluctuations in motivation. We investigated the effect of symbolic reinforcers on behaviors related to motivation during a token reinforcement learning (RL) task, using a novel RL model and data from five monkeys. We designed a state-based model that can capture reward-predicting features and produce state values at subtrial resolution. Our findings suggest that the value of a task state can affect willingness to initiate a trial, speed to choose, and persistence to complete a trial. Our model makes testable predictions for within-trial fluctuations of neural activity related to values of task states.

## Introduction

In most decision-making contexts, the objective is to maximize rewards and minimize punishments over time. In some situations, rewards are symbolic, such as money or points, in which case they can be exchanged for primary rewards, such as food or drink, in the future. Past studies have shown that animals and humans will work for symbolic reinforcers and symbolic reinforcers can drive learning and therefore motivate behavior (Hackenberg, 2009, 2018).

Motivation is a process that invigorates behavior in the present to reach rewards in the future (Berridge, 2004; Berke, 2018; O’Reilly, 2020). Motivation can be studied in the context of reinforcement learning (RL). Learning builds predictions of choice outcomes that can be used to direct future behavior (Sutton and Barto, 1998). *N*-Armed bandit tasks are often used to study RL in animals. These tasks can be modeled with Rescorla–Wagner (RW)-RL models (Recorla and Wagner, 1972), because the choices lead probabilistically, but immediately, to a primary reinforcer (Bartolo and Averbeck, 2020; Beron et al., 2022). However, one can also use symbolic reinforcers, for example, tokens or money, to drive learning (Jackson and Hackenberg, 1996; Kirsch et al., 2003; Seo and Lee, 2009; Delgado et al., 2011; Taswell et al., 2018, 2021, 2023; Falligant and Kranak, 2022; Yang et al., 2022). In these tasks, subjects learn to make choices to obtain tokens, which are predictive of primary reinforcers in the future. Token-based learning tasks set up a distinction between two types of cues that predict rewards in different ways. Specifically, tokens predict rewards on long-time scales, deterministically, whereas choice cues predict tokens. Furthermore, the relation between cues and tokens must be learned. Such tasks involving symbolic reinforcement cannot be accurately captured with current RL models, because the distinction between rewards and symbolic reinforcers cannot be made explicit.

Thus, we developed a Markov decision process (MDP) model to characterize the value of symbolic reinforcers and the computational mechanism that links cues through tokens to rewards. The MDP not only allows us to account for choice behavior (similar to existing stateless models) but also gives us an additional explanatory power in three ways. First, the MDP allows us to examine the effects of task factors that are independent of learning, like the number of accumulated tokens, on motivation. The RW model does not incorporate these factors. Second, it allows us to jointly model multiple factors that are predictive of reward in a single integrated term, the state value. Because the MDP is state-based and trials can have multiple states, the state value can then be related to behavioral and neural changes with subtrial resolution. Typical RL models have value estimates that only update trial by trial based on the chosen value. Third, the MDP framework also allows for examination of how each feature contributes to the state value while accounting for nonlinear interactions between multiple features. To establish the validity of the model to account for motivational processes, we examined the relationship between the state value (i.e., the expected discounted sum of future rewards) and behavioral measures associated with motivation. Five monkeys performed a task where they learned to select images that led to gaining or losing tokens. The tokens were cashed out every four to six trials, one juice drop per token. To examine the ability of the model to capture motivation, we conducted regressions between the state value and change in the state value and three behaviors linked to motivation. To demonstrate the effect of each task dimension included in the model, we performed marginalization analyses, where each feature was removed and the analyses were repeated. These analyses demonstrated that all features that drove value in the model contributed to motivation. Taken together, our results make predictions for how neural activity might evolve in RL circuits during a task involving symbolic reinforcement.

## Materials and Methods

### Subjects

The subjects included three male and two female rhesus macaques with weights ranging from 6 to 11 kg. Four monkeys were used as control monkeys in a previous study (Taswell et al., 2018). One additional monkey was a naive monkey whose first task was the tokens task. For the duration of collecting behavioral data, monkeys were placed on water control. On testing days, monkeys earned their fluid from performance on the task. Experimental procedures for all aspects of the study were performed in accordance with the *Guide for the Care and Use of Laboratory Animals* and were approved by the National Institute of Mental Health Animal Care and Use Committee.

### Experimental design

We conducted analyses on previously published data (Taswell et al., 2018) and data from one additional subject. We use data from one variant of the tokens task, previously called Stochastic Tokens with Loss (referred to as TkS).

The images used in the task were normalized for luminance and spatial frequency using the SHINE toolbox for MATLAB (Willenbockel et al., 2010). Image presentation was controlled by personal computer-running Monkeylogic toolbox (Version 1.1) for MATLAB (Asaad and Eskandar, 2008; Hwang et al., 2019). Eye movements were monitored using the Arrington ViewPoint eye-tracking system (Arrington Research).

### Stochastic tokens task with gains and losses

**Figure 1.**
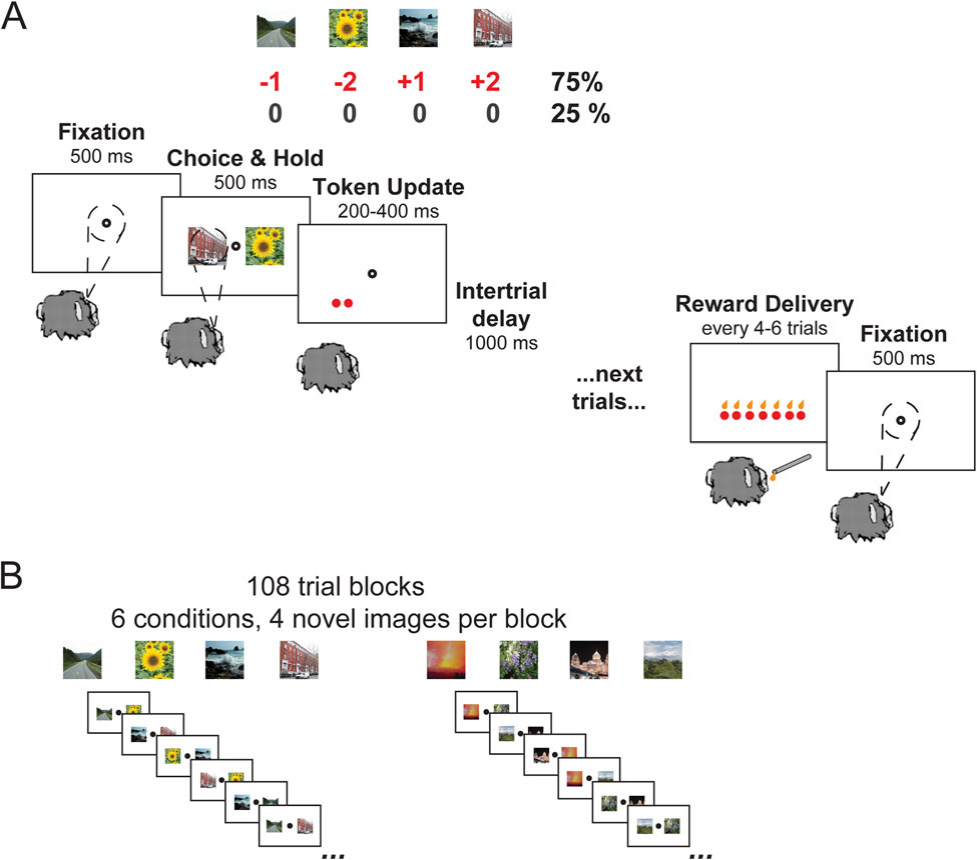
An overview of the tokens task with stochastic rewards. ***A***, The flow of a single trial. First the monkey fixates on the screen and is required to hold fixation for 500 ms. Two cue images appear on either side of fixation, each of which is associated with gaining or losing tokens (+2, +1, −2, −1). The monkey must make a saccade to one of the images and hold their gaze for 500 ms. After a successful hold, the NTk associated with the chosen option appears on the screen 75% of the time, and in 25% of the time, nothing changes. After a 1,000 ms intertrial delay, the next trial begins. Every four to six trials, one juice drop was given for each token that had been accumulated, as tokens were simultaneously removed from the screen. The monkey started the subsequent trial with zero tokens. ***B***, The monkeys learned through trial and error which visual images were associated with gaining tokens and which images were associated with losing tokens. Four new images were presented every block of 108 completed trials. Each pair of cues (6 in total) was seen 18 times (9 left/right, 9 right/left). Image credit: Wikimedia Commons (scene images).

Blocks consisted of 108 trials that used four novel images that had not been previously presented to the animal. Each image was associated with a token outcome (+2, +1, −1, −2), such that if that image was chosen, the animal gained or lost the corresponding number of tokens (NTk) 75% of the time and received no change in tokens 25% of the time (Fig. 1*A*). On each trial, monkeys had 2,000 ms to acquire a fixation spot at the center of the screen and were required to hold fixation for 500 ms. After monkeys held the central fixation, two of the four possible images would appear to the left and the right of the fixation point. The animal had 1,000 ms to choose one of the images by making a saccade to an image and holding their gaze on the image for 500 ms to indicate their choice. If the monkey moved their eyes outside the fixation window during fixation, did not choose a cue, or did not hold the cue long enough, the trial was aborted and repeated immediately. All trials were repeated immediately until they were completed before a different cue pair was shown. After a successful hold of gaze on a choice, tokens associated with the image were then added or subtracted from their total count, represented by circles at the bottom of the screen. Note that the animals could not have fewer than zero tokens. After an intertrial interval (ITI) of 1,000 ms, the next trial would begin with the accumulated tokens visible on the screen the entire time. Every four to six trials, tokens were cashed out such that one token disappeared at a time and one drop of juice was delivered. During this cash-out epoch, one drop of juice was delivered and one token disappeared, until all tokens were gone. The animal did not choose when to cash out; rather the probability of exchanging tokens for juice drops was a uniform distribution over four to six trials.

There were six cue conditions in the task, defined by the possible pairs of the four images. The conditions within a block were presented pseudorandomly, such that the animals saw each condition twice (same images, opposite sides) every 12 trials before seeing any condition a third time. This prevented strings of trials with loss versus loss that could lead to aberrant behaviors. At the end of each 108-trial block, we introduced four new images, and the animals restarted learning associations between the pictures and the token outcomes (Fig. 1*B*). The animals completed approximately nine blocks of images per session, and ∼20 sessions from each animal were used for subsequent analyses.

### Model framework

We modeled the tokens task using a partially observable Markov Decision Process (POMDP). To fit the POMDP to each animal's behavior, we leveraged the RW-RL model to calculate the average values of each of the four cue images and to verify the validity of our MDP results for fitting choice probability curves across the cue conditions. Details of this process are in the following sections.

### RW-RL model

We used a variant of the RW-RL model as was previously used to model the tokens task (Taswell et al., 2018).

We fit a Rescorla–Wagner value update equation given by the following:

(1) $$v_{i}(t+1)=\;v_{i}(t)+\alpha_{i}(R-v_{i}(t)),\;(1)$$

where the variable $v_{i}$ is the value estimate for cue option *i* that was chosen on trial *t*, *R* is the change in the NTk that followed the choice in trial *t*, and $\alpha_{i}$ is the cue-dependent learning rate parameter. In past work on this data, the model with a separate learning rate parameter for each cue was found to be the best RW-RL model fit to the data. Thus, we continued using this formulation of the RW-RL for these analyses, although the results described in this study are not contingent on this choice.

The values computed in Equation 1 were then used to compute choice probabilities for each cue pair using the softmax function as follows:

(2) $$d_{j}(t)=(1+\;e^{\beta_{\mathrm{RW}}(vi(t)-vj(t))}{)}^{-1},\;\;\;d_{i}(t)=1-d_{j}(t),\;(2)$$

where *β*_RW_ is the consistency choice parameter, fit across all six cue conditions, and *i* and *j* are the two choice options. We then maximized the likelihood of the animal's choices, *D*, given the parameters, using the cost function as follows:

(3) $$f(D|\alpha_{i},\beta_{\mathrm{RW}})=\;\underset{t}{\prod }\;[d_{1}(t)c_{1}(t)+d_{2}(t)c_{2}(t)],\;(3)$$

where *d*_1_(*t*) is the choice probability value for Option 1 on trial *t* and *c*_1_(*t*) and *c*_2_(*t*) are indicator variables that take on a value of 1 if the corresponding option was chosen and 0 otherwise. This model was fit across blocks in each session for each monkey to give one set of fit parameters for each session.

**Figure 2.**
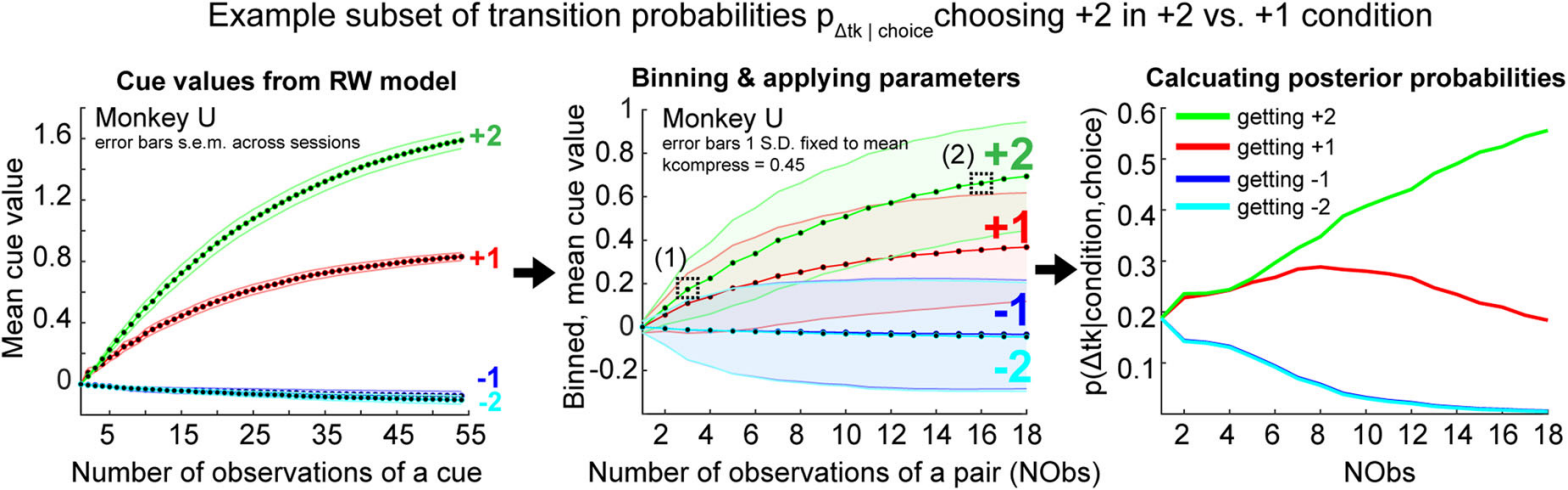
Mean cue values extracted from the RW-RL model and transition probabilities for change in tokens given a cue image selection. (Left) Mean cue values are plotted for NObs, for each of the four cues in the stochastic tokens task (error bars indicate SEM across blocks for Monkey U). The +2 cue curve is the largest mean value, followed by the +1 curve, which reflects how the animals learned to select the +2 and +1 image cues. The −2 and −1 curves are similar to each other and are slightly negative, which reflects that the animals did not learn the value differences between the −2 and −1 cue options and did not select them often when they were shown. The mean values were extracted from the RW-RL model fits to each monkey. (Middle) Example set of curves from Monkey U with scaling parameter (*k*_compress_) applied to the mean cue values (see Materials and Methods, MDP model, and Eq. 9). Each data point is an average of the three conditions in which the cue was observed to reduce the number of overall states required to track learning; instead of tracking 54 times a cue was observed, the model tracks the 18 times a cue pair was shown. For example, for the +2 curve, a single data point would be the average value of the +2 cue value from the conditions +2 versus +1, +2 versus −2, and +2 versus −1. (1) The dashed line box highlights +2 cue value for early in learning at trial 3. (2) Dashed line box highlights +2 cue value late in learning at trial 16. Error bars are the mean standard deviation that was fixed across all cues. This mean variance (*σ*) was used in the posterior probability calculation (Eq. 7). (Right) A subset of transition probabilities are plotted as a function of NObs. These transition probabilities were derived from the mean value curves for the +2 versus +1 condition and choice of +2 for each possible token outcome. *p*(Δtk = 0 / choice +2 cue) is always 0.25 and is not shown.

Mean cue values as a function of learning trial in each block (from the RW-RL model) were used to generate transition probabilities for the MDP. This is discussed in detail below. To extract mean cue values, we averaged all $v_{i}$ for a single cue across sessions. This produced four curves that reflected the change in the cue value across trials for each monkey (Fig. 2, left). These cue values were then used to produce a set of transition probabilities, used in Equation 4 to compute MDP state values. In the next section, we describe the general construction of the MDP and all of its transition probabilities. We then cover how the RW model was used to produce a small set of key transition probabilities related to learning (Fig. 2 right, middle).

### MDP model

The MDP model computes the value of each task state and, for states with actions available, chooses the action with the maximum value. Task states were defined by four features of the task: NTk, trials since cash-out (TSCO), task epoch (TE), and number of observations of a cue pair (NObs). The state space included all possible combinations of these features across a single block of trials, such that the bounds for each feature were as follows: NTk, 0–12; TSCO, 1–6; TE, 1–10 (which included fixation, six cue conditions, token outcome, cash-out, ITI); and NObs, 1–18. Using NObs as a feature allowed us to avoid having to track each time a cue was shown (54 times per block), chosen and rewarded across trials, and to reduce the size of the state space by 18^12^ states, which also made a tabular form of the model tractable. The model of the task was in epoch time (i.e., event based), rather than true time (i.e., seconds based).

The state space can be considered as a graph with edges and nodes, where the states (nodes) are defined by the possible combinations of these features, and the edges are the transitions to future states. A trajectory through the state space represents one trial and as the model proceeds through a block, the trajectory traverses through the state space.

The state value, sometimes called state utility, $u(s_{t})$, was calculated using the following equation:

(4) $$u(s_{t})=\;\underset{a_{t}\in A}{\max}\;\left[\;r(s_{t},\;a_{t})+\;\gamma \underset{\;j\in S_{t+1}}{\sum }\;p(\;j|s_{t},\;a_{t})u(\;j)\right],\;(4)$$

where *s_t_* is the state, *a_t_* is the action taken at that state, $u(s_{t})$ is the state value, $r(s_{t},\;a_{t})$ is the immediate reward, *γ* is the discount factor, $p(\;j|s_{t},\;a_{t})$ is the transition probability to future state *j*, and *S_t_*_ + 1_ is the set of immediate future possible states from state *s_t_* if one takes action *a_t_*. The summation over future states (*j*) is the expected future utility, taken across the transition probability distribution $p(\;j|s_{t},\;a_{t})$. The transition probabilities were given by the task design [e.g., *p*(cash-out) = 0 on Trial 1], whereas transition probabilities for the token update were derived from RW values (Fig. 2). A range of discount factors (*γ* = 0.8, 0.85, 0.9, 0.95, 0.99, 0.999) were tested. Because *γ* = 0.999 produced the least error for the regressions and for all monkeys, we chose to use this for all subsequent analyses and did not explore fitting the discount factor as a separate parameter. The exact value of the discount factor, however, had minimal effect on our regressions with behavior. We used value iteration to fit the MDP (Puterman, 2014). The algorithm loops over all possible states and recomputes Equation 4 until both the policy and state values converged. This took ∼100 iterations across the state space for each MDP that was fit.

The transitions between states [i.e., $p(\;j|s_{t},\;a_{t})$] include fixation to the six cue states, cue state to token update, token update to cash-out or ITI, and ITI to fixation. The transition probabilities from fixation to any cue state were modeled as *p*_cues _= 1/6 as there were six possible cue pairs. The transition probabilities from token update to cash-out were *p*_cash-out _= 0 for TSCO 1–3, *p*_cash-out _= 0.33 for TSCO 4, *p*_cash-out _= 0.50 for TSCO 5, and *p*_cash-out _= 1.0 for TSCO 6. Transition probabilities for the transition to a change in tokens given a cue image selection, i.e., *p*(change in tokens / image choice in a given condition), were fit using the behavioral data and average cue values that were extracted from the RW-RL model fits. Thus, this is not an ideal observer estimate, but rather our inference of the monkey's estimate. An ideal observer would learn the cue values in a single exposure to the cue outcomes due to the equation that defines state value (Eq. 4). Once utility differences emerge, the model will always pick the larger option. However, the monkeys take longer than one trial to learn the value differences. Thus, we matched the MDP learning to monkey choice behavior. To do this, we developed a method to extract estimates of probability distributions over token outcomes that more accurately reflect monkey choices than optimal behavior. These transition probabilities represent the monkey's mapping of individual cues to outcomes, e.g., which picture leads to +2 tokens. The cue values are related to this mapping, thus making the RW-RL values an approximation to the process by which the animal learns the outcomes related to each cue image (Fig. 2).

We had to infer the monkey's estimate of the NTk they would receive when they chose a given option in order to accurately model choice behavior. For example, in the first trial of a new block, the monkeys had no experience with any options, and therefore they should assume that the choice of any option could lead to either −2, −1, 0, 1, or 2 tokens. However, after 10 trials the monkeys had a reasonable estimate of the token outcomes associated with each option. This process makes the MDP have partially observable states, as we estimate the transition probabilities using mean values from the RW-RL model. We carried out this estimate in two steps. First, we calculated the value estimates for each option as a function of the number of times they had seen each option, from 1 to 54 times (Fig. 2, left). We then collapsed this into each time they had seen a condition by averaging across the three trial-by-trial values to produce the value estimates as a function of the number of times they had seen each cue pair, from 1 to 18 times (Fig. 2, middle). We then used these estimates to calculate the posterior probability that the choice of a given cue would lead to a given outcome (i.e., Δtk). For the outcome of no tokens, p(Δtk=0)=0.25. For all other possible outcomes, the following equations were used:

(5) $$p(\Delta \mathrm{tk}=\{+2,+1,-2,-1\})=\frac{0.75}{4},\;(5)$$

(6) $$p(\Delta \mathrm{tk}|v_{\mathrm{cue}})=\frac{\;p(v_{\mathrm{cue}}|\Delta \mathrm{tk})p(\Delta \mathrm{tk})}{\;p(v_{\mathrm{cue}})},\;(6)$$

(7) $$p(v_{\mathrm{cue}}|\Delta \mathrm{tk}=j)=\frac{1}{\sigma \sqrt{2\pi }}e^{\left(-\frac{1}{2}\;\frac{{\left(x_{\mathrm{cue}}-\mu_{j}\right)}^{2}}{\sigma^{2}}\right)},\;(7)$$

(8) $$p(v_{\mathrm{cue}})=\overset{4}{\underset{\;j=1}{\sum }}\;p(v_{\mathrm{cue}}|\;\Delta tk_{j})p(\Delta tk_{j}),\;(8)$$

where $v_{\mathrm{cue}}$ is the mean value of a single cue for a given NObs, Δtk=+2,+1,−1,−2 is extracted from the RW model (Fig. 2), x is the mean of the cue value of the chosen option, $\mu_{j}$ is the mean value of cue *j*, and *σ* is the standard deviation of the cue values. Transition probabilities were calculated for all possible choices and NObs and were not dependent on other MDP features such as NTk or TSCO. For example, three trials into the block, mean cue values were 0.17, 0.11, −0.01, and −0.01 for the +2 cue, +1 cue, −1 cue, and −2 cue, respectively (Fig. 2, middle). To calculate $p(\Delta \mathrm{tk}=+2|v_{\mathrm{cue}}=2)$ (i.e., the probability of receiving two tokens for choosing the +2 cue), x=0.17, $\mu_{1}=0.11$, $\mu_{2}=-0.01$, and $\mu_{3}=-0.01$, which produces $p(\Delta \mathrm{tk}=+2|v_{\mathrm{cue}}=2)=0.24$ in the +2 versus +1 condition. Later in the block, for example, on NObs = 16, mean cue values were 0.66, 0.36, −0.03, and −0.04 for the +2 cue, +1 cue, −1 cue, and −2 cue, respectively. At this point in learning, $p(\Delta \mathrm{tk}=+2|v_{\mathrm{cue}}=2)=0.52$ in the +2 versus +1 condition.

The mean cue values were used to fit the set of transition probabilities *p*(Δtk / choice) for Δtk = 0, 1, 2, −1, and −2 and choice = cue 1 and cue 2. First, an MDP was fit using the mean cue values for each animal in order to compute a converged policy of choices and action values without any free parameters. These MDP models captured general trends of behavior to select the better options (i.e., an optimal MDP) but showed faster learning than the animals learned. To better match animal learning behavior, we optimized the transition probabilities underlying the behavioral performance of the MDP.

To optimize the set of transition probabilities *p*(Δtk / choice), mean cue values were used with two free parameters, (1) a scaling parameter for the mean cue values as follows:

(9) $$v_{\mathrm{cue}}^{s}=k\;*\;v_{\mathrm{cue}},\;(9)$$

for all mean cue values, and (2) an inverse temperature parameter for the choice probability (*β*^MDP^). In addition, the variance, *σ* (Eq. 7), of the mean value curves for each animal was set to the average variance across all four cues, allowing variance to vary across trials, but not across cue values. Using the initial MDP fit and the behavioral data from the task for each animal, the two free parameters were fit jointly by minimizing the root mean squared error between the MDP choice probability and average performance across sessions for each monkey (Table 1). The resulting parameters and transition probabilities were then used to refit the MDP until the state values and policy reconverged. In addition, MDP models were fit using a range of discount factors (*γ* = 0.8, 0.85, 0.9, 0.925, 0.95, 0.99, 0.999) for each animal's dataset. To determine the discount factor that produced the best fit to behavior, we used each *γ* to regress on fixation reaction times (RTs), choice RTs, and *p*(Abort) and to produce choice probability curves for the six conditions (see below, Regressions and statistical analyses, for details on the regressions). For all monkeys, *γ* = 0.999 produced the best fits to these behavioral data metrics, and thus, *γ* = 0.999 was used for all models.

**Table 1.** MDP free parameter values for each monkey

Monkey	Mean value scaling parameter (*k*)	Choice probability parameter (*β*^MDP^)
Monkey U	0.45	2.77
Monkey B	0.71	1.87
Monkey S	1.04	1.15
Monkey P	1.14	1.34
Monkey A	0.53	2.24

Two parameters were optimized to minimize the error between the MDP choice probability and the monkey's choice behavior. The mean value scaling parameter acted as a scalar on the variance of the value curves (Eq. 9). The choice probability parameter was the inverse temperature parameter used to calculate the choice probability using action values derived from the MDP.

### Regressions and statistical analyses

The comparison of performance of the MDP and RW models at predicting choice behavior was conducted using a comparison of correlation coefficients. Correlation coefficients (*r*_1_, *r*_2_) were calculated between the average choice behavior and each model separately. The values were then Fisher *z* transformed to compute a *p* value for a two-sided test for differences between the correlation coefficients.

State values were extracted for all trials and epochs using the MDP fits for each animal. This produced a table of states such that the value of each state was $u(s_{t})=\;f(\mathrm{NTk},\;\mathrm{TSCO},\;\mathrm{TE},\;\mathrm{NObs})$. These state utilities were used to characterize trial-by-trial relationships to RT to acquire fixation, choice RT, and trial aborts.

Mean RTs were computed by averaging RTs across blocks of trials and then averaging across sessions for each monkey. Scatter plots of RTs from individual sessions do not include outlier RTs. Outlier RTs were removed using Tukey's method, RT > q0.75 + 1.5*IQR and RT < q0.25–1.5*IQR, where IQR is the interquartile range.

To assess the relationship between the state value and RTs to acquire fixation, the linear regression on the state value was performed as follows:

(10) $$\log(RT_{\mathrm{acquire}\_\mathrm{fixation}})=\;\beta_{0}+\;\beta_{V_{\mathrm{Fix}}}V_{\mathrm{fix}},\;(10)$$

where $V_{\mathrm{fix}}$ is the value of the fixation state. RTs were log-transformed before the regression.

To assess the relationship between the state value and RT to choose, linear regression on the state value was performed as follows:

(11) $$\log(RT_{\mathrm{acquire}\_\mathrm{fixation}})=\;\beta_{0}+\;\beta_{V_{\mathrm{cue}}}V_{\mathrm{cue}}+\;\beta_{\Delta V}(\Delta V),\;(11)$$

where $V_{\mathrm{cue}}$ is the value of the cue presentation state and $\Delta V=V_{\mathrm{cue}}-V_{\mathrm{fix}}$. RTs were log-transformed before the regression.

To assess the relationship between the state value and the probability of aborting a trial, logistic regression on the state value was performed as follows:

(12) $$p(\mathrm{Abort})=\;(1+\;e^{-(\beta_{0}+\;\beta_{V_{\mathrm{cue}}}V_{\mathrm{cue}}+\;\beta_{\Delta V}(\Delta V))}{)}^{-1},\;(12)$$

where $V_{\mathrm{cue}}$ is the value of the cue presentation state and $\Delta V=V_{\mathrm{cue}}-V_{\mathrm{fix}}$. We additionally assessed the effect of cue condition on *p*(Abort) using a mixed-effect ANOVA, where the monkey was the random effect and cue condition was the fixed effect.

To assess whether regressors were significantly different than zero, for each animal, *t* tests on the distributions of *β* values across sessions for each regressor were performed for each animal. These values are reported in the text. Mean parameter values did not appear to be Gaussian-distributed across monkeys. Therefore, to assess whether the regressors were significantly less than zero at the group level, the nonparametric Wilcoxon signed-rank test was used on the distribution of mean parameter values across animals. Results of these tests are reported in the figure captions and text. To show group trends in relationships between RTs and regressors, we conducted 1D kernel smoothing on each monkey's data with σ=0.5 with a Gaussian kernel.

We also sought to examine the contributions of the different variables that defined the state to each regression by marginalizing over one factor at a time, to remove its effect, and carrying out the correlation analyses. To perform this marginalization, we averaged over the state values for all possible values of a single feature, given the other features. For example, to compute state values for all features averaging over NTk, with TSCO=4,TE=1,NObs=12, we computed $V_{\mathrm{fix}}=\mathrm{mean}\;(\;f(\mathrm{NTk}=0:8,\mathrm{TSCO}=4,\mathrm{TE}=1,\mathrm{NObs}=12))$, as NTk = 8 is the maximum NTk possible when TSCO = 4.

For additional regressions that were conducted directly on MDP features and chosen value from the RW model, multivariate linear or logistic regressions were used when relevant. Each regression was performed separately for each session of data for each animal. Regression coefficients were reported as mean regression values across animals with error as standard error of the mean (SEM) across animals. Statistical testing was performed at the group level using the nonparametric Wilcoxon signed-rank test. For RT to acquire fixation, the linear multivariate regression included NTk, TSCO, and NObs. For RT choice, two multivariate linear regressions were performed, one on chosen value extracted from the RW model and one on NTk, TSCO, NObs, and cue condition (TE 2–7) as a categorical variable. RW chosen value was computed for each trial by using the model-derived value for the option chosen on each trial. This was then used for regressions instead of state value or state value features. For probability of aborting a trial, a multivariate logistic regression was computed using the features NTk, TSCO, NObs, and cue condition (TE 2–7) for trials where aborts happened after cue onset. For abort trials that happened during fixation (i.e., before cue onset), features NTk, TSCO, and NObs were used.

### Code accessibility

All code used to generate the results in this manuscript can be accessed on GitHub here: https://github.com/dcb4p/mdp_tokens.

## Results

We designed a novel MDP model to capture fluctuations in state values with subtrial resolution in a task with symbolic reinforcers. We then examined the relationships between changes in state values and behaviors related to motivation. First, we describe the tokens task, which involved symbolic and primary rewards. We then give an overview of the model design which accounts for features that predict reward and how these features were incorporated into states. We then go through three behaviors, acquiring fixation, choice RTs, and aborting trials, and demonstrate how changes in the state value are correlated with these behaviors that have been previously linked to motivation.

Five monkeys were trained on a stochastic tokens task (Taswell et al., 2018; Fig. 1). Briefly, each block of the task used four novel images, and the choice of each image led to a different possible token outcome (+2, +1, −1, −2). In each trial, two of the four images were presented as options, and the monkey made a saccade to one of the cues and held their gaze to indicate their choice. After the choice, the monkey received, stochastically, the corresponding change in tokens on the screen. In 75% of the trials, they received the NTk associated with the cue, and in 25% of the trials, the NTk did not change. Every four to six trials was a cash-out trial. In cash-out trials, the monkey received one drop of juice for each accumulated token. The monkey would then start over, accumulating tokens until the next cash-out trial. Each of the pairs of cue images (six in total) was presented 18 times during a block of trials. At the end of the block, the four cue images were replaced with novel images, and the monkey restarted learning the associations between the images and token outcomes. Behavioral performance and learning were assessed for each animal by the increasing frequency with which the monkeys chose the image associated with the better option in each condition over the course of a block.

In this task the monkeys were not given rewards on every trial. Rewards were only given at the time of cash-out, which was every four to six trials. Commonly used RL models, such as the RW model (Sutton and Barto, 1998), do not make a distinction between symbolic reinforcers and primary rewards, and therefore they do not have a natural way to model the difference between primary and secondary reinforcers. To address this, we developed a state-based MDP model of the stochastic token RL task to capture the relevant features of the task that would affect motivation and choice behavior. With such a model, we can not only account for choice behavior but also test hypotheses regarding other motivated behaviors, through the lens of state value. Within MDP models, values and available actions are defined by the current state. In our model, the state is a function of the NTk, TSCO, TE, and NObs. The state space consists of all possible combinations of these four features. The model, once trained, has states that inherit value from their proximity to the true rewarding state (cash-out), similar to how a well-trained monkey expects to earn juice in the future.

State values are given by the maximum action value in each state. State values and action values are equivalent in all epochs except the choice epoch, because only one action is possible in the other epochs. Rewards are only delivered in the cash-out period, and therefore immediate expected values, *r*(*s_t_*, *a_t_*), are 0, except in the cash-out period when more than zero tokens have been accumulated. In all other states, immediate expected values are 0, and state values are future discounted expected values, all of which are filtered through the graph from the cash-out period. Thus, future expected values in each state follow from the features of the task that predict the delay to and size of the reward that will be delivered in cash-out. While some states only have one possible action (e.g., ITI and fixation), these states are modeled to incorporate additional time delays into the model. Although we could have explicitly modeled the time, this would have resulted in a large number of additional similar states. By modeling these states, we can ask about RTs to acquire fixation. Additionally, in future work we will study the effects of value on neural activity during ITIs with different numbers of tokens.

**Figure 3.**
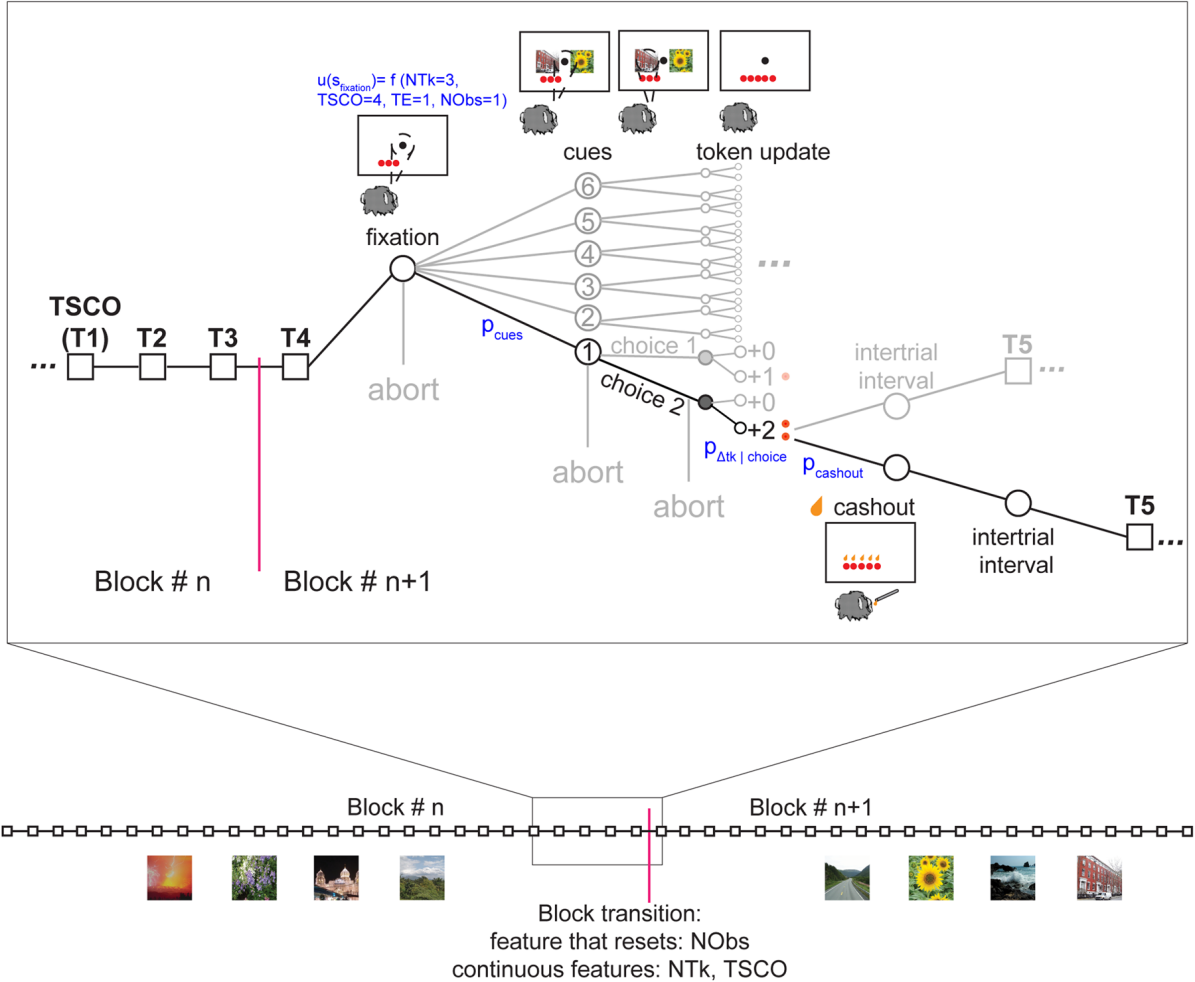
An overview of the task state space for the MDP framework. Each square node represents a trial within a block. The subset of trials expanded shows a transition between blocks where the feature NObs is reset, while the other MDP features continue across blocks. Each circular node represents a state given by a set of task features: token count (NTk), TSCO, TE, number of cue condition observations (NObs). Each edge of the graph represents a transition probability or the transition between states that could be deterministic (*p* = 1) or probabilistic (*p* < 1). A single trial is highlighted, and the progression from each step in the task is shown for an example trial that ends in a cash-out. The example trial starts at fixation, and the monkey has three tokens, making the value of the state *f*(NTk = 3, TSCO = 4, TE = 1, NObs = 1). The transition probability *p*_cues _= 1/6 and represents the probability of any of the six cue conditions being the next state. At the cue states, the monkey must make a choice, and the model captures this choice policy behavior. The next set of states after the cue state is the outcome state; the transition is governed by the transition probability *p*_Δtk|choice 2_. In the example, the monkey receives two tokens, and the model receives two tokens. After the outcome state, the monkey can transition to the ITI or cash-out; the transition to this state is governed by *p*_cash-out_. During cash-out, one drop of juice was given for each token as tokens disappeared from the screen. All of the tokens disappear before proceeding to the next ITI and subsequent trial. Image credit: Wikimedia Commons (scene images).

For example, the value of the fixation state is a combination of the NTk accumulated, TSCO, and NObs witnessed up until that trial (Fig. 3). The value of the fixation state is the sum of the immediate expected value (which is 0 at fixation because no juice is ever delivered) and the future expected value, which is the expected value of the next state (i.e., the average over the cue states, each of which occurs with a probability of 1/6). Thus, the state value in the fixation state is inherited from the values of the cue states.

In each cue state, there is a choice between the two cues, which then leads to the token outcome states. As five of the six possible cue conditions have gain outcomes (once the monkey learns to select the better option), the state values of the cue conditions reflect this possible gain as it develops with learning. In the outcome states, the monkey can receive +2, +1, 0, −1, or −2 tokens. As there is also no immediate reward available during the cue states, state value comes from the future expected value of the ITI or cash-out states.

The monkey learns to choose options that maximize gaining tokens over losing tokens. We modeled this learning as an inference over the token outcome distribution associated with each choice, using a parameterized function of the number of times an option had been chosen. The model, which generated an estimate of the transition probabilities from cue to token outcome (*p*_Δtk|choice_), was fit to each monkey's choice behavior. Unlike the transition from the fixation state to the cue state (*p*_cue_), the probability of transitioning to each outcome state (*p*_Δtk|choice_) changes as the monkey learns the associations between the cue options and token outcomes. For example, at the start of a block, the monkey does not know which cue image predicts which token outcome, and *p*_Δtk = +2|choice _= *p*_Δtk = +1|choice _= *p*_Δtk = −2|choice _= *p*_Δtk = −1|choice_. Once the monkey learns which cue image is associated with +2 tokens, they are more likely to select that option, and we assume they infer that the probability of getting two tokens, *p*_Δtk = +2|choice_, is larger given the choice of that option. Thus, the transition probabilities change over the course of a block as the number of times the cue pair has been observed (NObs) increases. The changes in these transition probabilities reflect learning (Fig. 2, middle , right).

At the time of token outcome, the next possible states are either the ITI or cash-out, governed by the transition probability *p*_cash-out_. This transition is a feature of the task and does not change with learning. At the time of cash-out, the state value is the sum of the immediate reward (one drop of juice per token present) and the next state, which is the ITI, with zero tokens. In the model, this means the state value will drop after cash-out if the monkey cashed out tokens for juice, as the state value depends on the NTk.

### Changes in task features drive fluctuations in the state value

**Figure 4.**
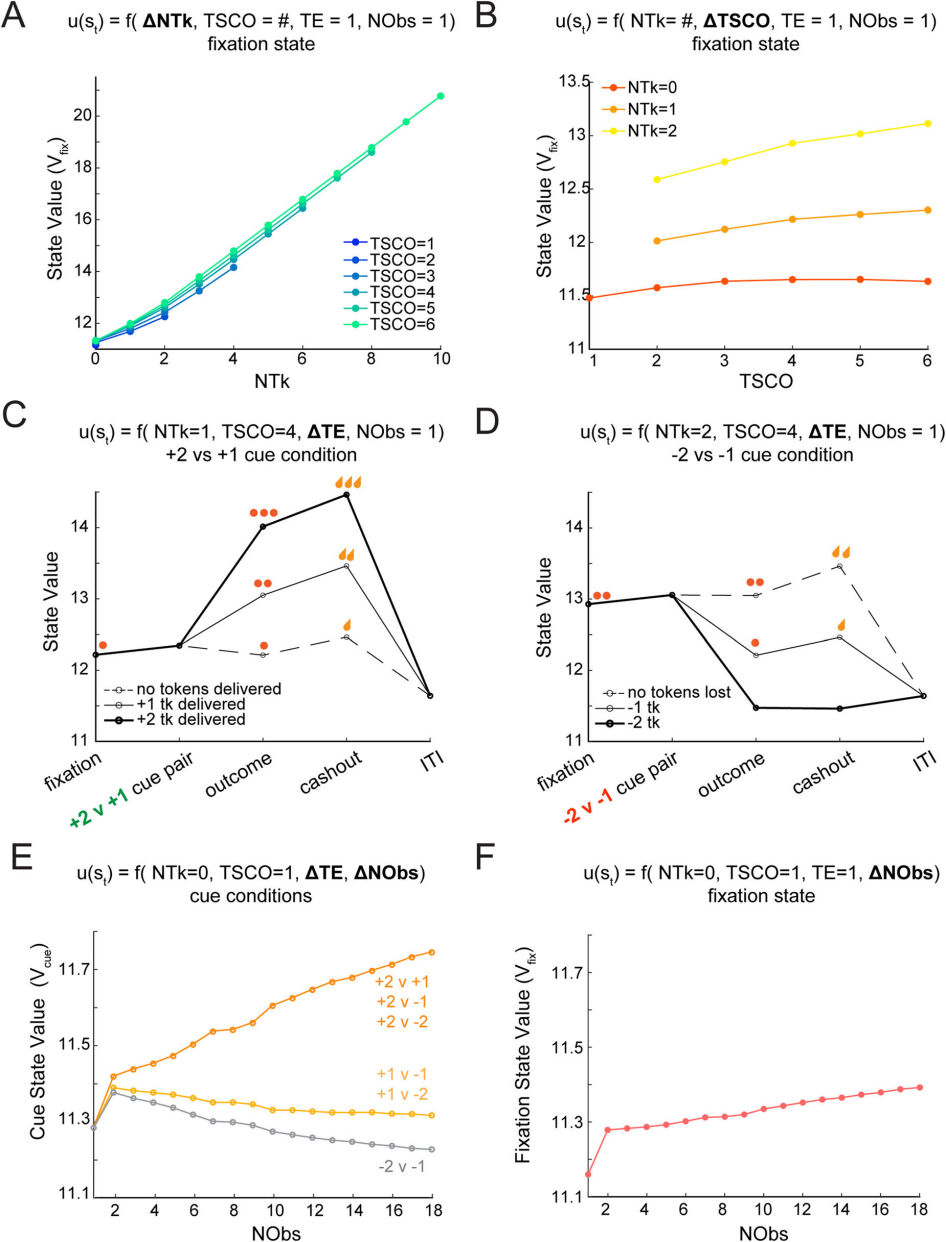
How changes in state features can affect the state value (an example from Monkey B). ***A***, Fixation state value (*V*_fix_) versus token count (NTk). As NTk increases, the state value increases. For one trial since cash-out (TSCO = 1), only one trial has been completed, so the maximum NTk available is two. As the TSCO increases, the juice will be delivered sooner, which is reflected by a higher state value. ***B***, Fixation state value (*V*_fix_) versus TSCO. As TSCO increases, the state value increases when there is more than one token (NTk = 1, NTk = 2). If there are zero tokens (NTk = 0), then the state value increases until TSCO = 4. When cash-out becomes possible, the state value begins to decrease when there are no tokens, because a cash-out epoch can occur, with zero juice delivery, resetting the interval before the juice can be delivered again. ***C***, State value versus TE for a single cue condition +2 versus +1. As the model proceeds through a trial, the state value changes depending on TE and the outcome. In this example, there is one token at fixation (red dot, far left), and three traces are shown, one to represent each possible outcome (+2 tokens, +1 token, 0 token change). The state value increases for gaining tokens and decreases slightly when no tokens are gained. The state value at cash-out corresponds to the token count. The state value is identical for all three outcome traces during the ITI after a cash-out. ***D***, State value versus TE for a single cue condition −2 versus −1. In this example, there are two tokens at fixation (red dots, far left), and three traces are shown, one to represent each possible outcome (−2 tokens, −1 token, 0 token change). The state value decreases when tokens are lost and stays constant when the outcome is zero tokens. At the time of cash-out, the state value depends on whether tokens are present. Like in ***C***, the state value is identical for all three outcome traces during the ITI after a cash-out. ***E***, Cue state value (*V*_cue_) versus NObs. As NObs increases, the state value increases for all cue conditions that include the best option (+2 tokens). The state value for the cue conditions with the +1 option decreases with learning and plateaus. The state value for the loss versus loss condition (−2 vs −1) decreases with NObs. ***F***, Fixation state value versus NObs. As NObs increases, the value of the fixation state increases. As the monkey proceeds through a block, they learn the associations between the cue images and token outcomes, and it is more likely the monkey will select the better options (+2 and +1). In the MDP, this means that as NObs increases, it will be more likely that tokens will be received, which causes an increase in the future expected value and thus state value.

After fitting each monkey's behavior with a separate MDP, we end up with state values for each animal for all states in the task. While on average, being closer to cash-out will lead to a higher state value, state values can change depending on how other features change. Here we discuss how state values change with variation of each state feature in a series of examples from a single monkey. In these examples, state values span the range of ∼11–20 for Monkey B. Each monkey had slightly different value ranges after fitting the MDP to their choice behavior but identical trends, as the trends are contingent on the architecture of the model and not the monkey's choices. For example, having more tokens increases the state value (Fig. 4*A*). Being closer to the cash-out state (e.g., TSCO > 4) also increases the state value (Fig. 4*B*). As the monkey proceeds through the TE (i.e., fixation, cue onset, outcome, cash-out, or ITI), the state value will also increase in conditions where tokens can be gained and, more subtly, because one is also getting closer to cash-out (Fig. 4*C*). For the −2 versus −1 cue condition where tokens can only be lost or maintained (if there is a no change outcome), the value of the state either decreases or increases marginally approaching cash-out (Fig. 4*D*). In the first trial of a block, when the monkey has started learning the associations between cues and outcomes (i.e., NObs = 1), the best option in a pair of cues will be ambiguous. This is reflected by identical state-action values in the model (Fig. 4*E*). Near the end of the block, when the monkey knows which cues correspond to +2 and +1 tokens (e.g., NObs 18), the state-action values will reflect the knowledge of the best option, and the state values at the time of the cue state will be higher for the conditions with +2 or +1 cues (Fig. 4*E*). Even though the fixation state precedes the cue state, the number of observations also affects the value of the fixation state and causes it to increase as NObs increases, because the monkey can make better choices when the options are presented (Fig. 4*F*). The minimum state value is at the baseline for all features, i.e., NTk = 0, TSCO = 1, TE = fixation, and NObs = 1 (Fig. 4*F*, NObs = 1).

The exact value of the baseline state value and the relationship between NObs and the fixation state value vary by model (i.e., monkey). This relationship is affected by three things: the token outcome transition probabilities, the discount factor, and the number of iterations for fitting the model. The discount factor was 0.999 for all monkeys, and the number of iterations for fitting each model was constant. Only the token outcome transition probabilities (*p*_Δtk|choice_), which were fit to each monkey's behavior, vary between monkeys in the models. Therefore, the larger the token outcome transition probabilities to gain outcomes, the larger the initial state value even at the time of fixation. In other words, when the monkey learned faster, these transition probabilities changed faster, and the state value increased faster with NObs.

### State-based MDP model of symbolic reinforcement captures learning behavior

**Figure 5.**
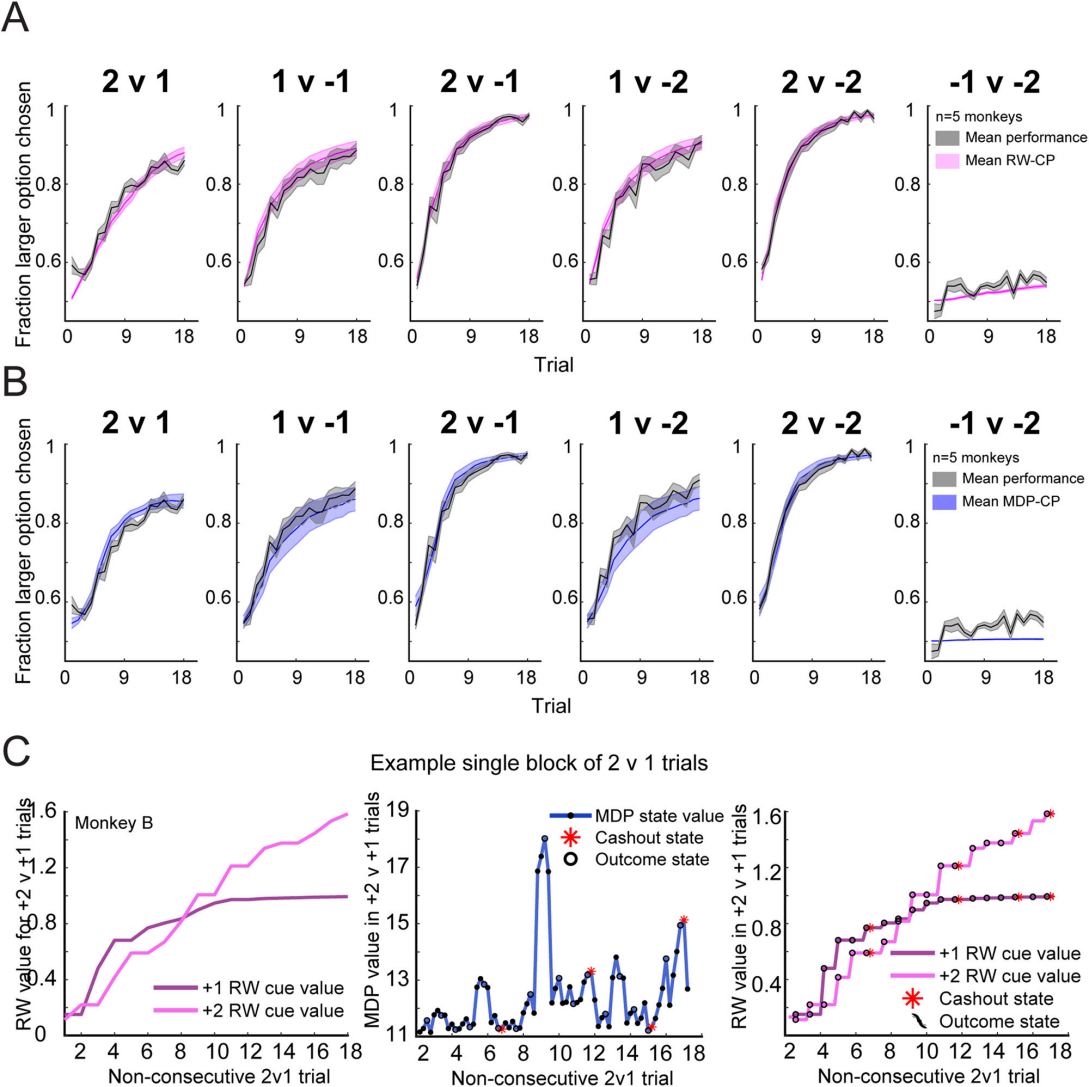
Validation of the MDP fits to choice behavior and illustration of the state-based model (MDP) versus stateless model (RW) changes in value. ***A***, Average performance and RW-RL (stateless) model fits for all subjects (*n* = 5) monkeys in the six task conditions (error bars indicate SEM across animals). ***B***, Same as ***A*** but average MDP (state-based) model choice probability instead of RW-RL model fits. ***C***, Example single block of 2 versus 1 trials demonstrating changes in the RW value for each cue (left), changes in MDP state values for identical trials and the states associated with each trial (middle), and the RW value extended across the same number of states, as if the RW model had states. In the middle panel, the peak around Trial 9 shows when the monkey had seven tokens and then experienced loss in subsequent interleaved trials that are not plotted. The MDP state values (middle) show a richer representation of fluctuations of value that are not captured by the RW model (left, right).

To test the validity of the choice policy of the MDP model for each monkey, we calculated the choice probabilities produced by the choice policy of each MDP, after passing action values through a softmax. The purpose of this was to validate that the MDP model could capture choices equally well to the RW model previously used to analyze choice behavior (Taswell et al., 2018, 2023). After fitting an MDP to each monkey, the choice probability was calculated using the action values for each choice in each cue condition, for each trial in a block (NObs 1–18). The action values were passed through a softmax with an inverse temperature parameter *β* (see Materials and Methods, MDP model), which controlled the stochasticity of the choice policy given two action values. An average choice probability across animals demonstrated that the choice probabilities produced by the MDP produced similar fits to the behavioral data to the RW-RL model, with no statistically significant difference between the correlation coefficients computed from the behavioral data and the two models (*r*_RW _= 0.9904; *r*_MDP _= 0.9868; difference in correlation, *p* = 0.25; Fig. 5*A*,*B*). Note that the detailed investigations of learning in the six task conditions is described in Taswell et al. (2018, 2023). Briefly, the monkeys chose the better option in five of the six conditions, and although they did not discriminate well between losses in the −2 versus −1 condition, the monkeys did learn to pick the smaller loss option statistically better than chance. This verified that the MDP captures choice behavior in the task, over and above its ability to model future state values. Further analyses using state values are, therefore, grounded in an accurate representation of choice behavior.

The purpose of comparing the MDP and RW models was not to suggest that the MDP captures choice behavior better than the RW model. The MDP model provides a richer view of changes in the value due to the incorporation of features that predict reward into states. As the RW model is stateless, value updates happen trial by trial and values for the option that is chosen more often will increase over the course of a block (Fig. 5*C*, left). In contrast, the MDP has four to five possible trial states (fixation, cue onset, outcome, cash-out, and/or ITI), and the value in a trial can be driven by several factors unrelated to learning but likely related to motivation (Fig. 5*C*, middle). The MDP can thus provide additional richness regarding fluctuations in predictors of reward, such as the NTk and the distance to cash-out. Even if the RW values are expanded in the same way across trials but are only updated at the time of the outcome (Fig. 5*C*, right), the RW values do not capture the same information that the MDP can capture.

With the calculation of state values, we can test hypotheses regarding each feature and the combination of features that predict the reward. Furthermore, we can test for fluctuations in the state value within trials and across trials, whereas the RW model only allows for value updates once per trial. In the MDP, TEs are separate states that can be affected by other features (NTk, TSCO, NObs). For example, the MDP can account for variation in the state value related to the proximity to the cash-out state. The NTk feature enables testing hypotheses related to how the value changes across trials and within trials as tokens are gained or lost. The MDP includes TSCO, which allows for testing hypotheses related to the distance from cash-out across trials. The NObs is accounted for with both the MDP and RW models. With the MDP, we can ask questions about how NObs affects state value in states other than the choice period, such as fixation and ITI.

The question then becomes whether the additional explanatory power has meaning for explaining behavior beyond choice behavior. To test this, we related MDP state values to three behaviors known to be linked to motivation: trial initiation, choice RTs, and trial completion.

### The time to acquire fixation is related to the value of the fixation state

**Figure 6.**
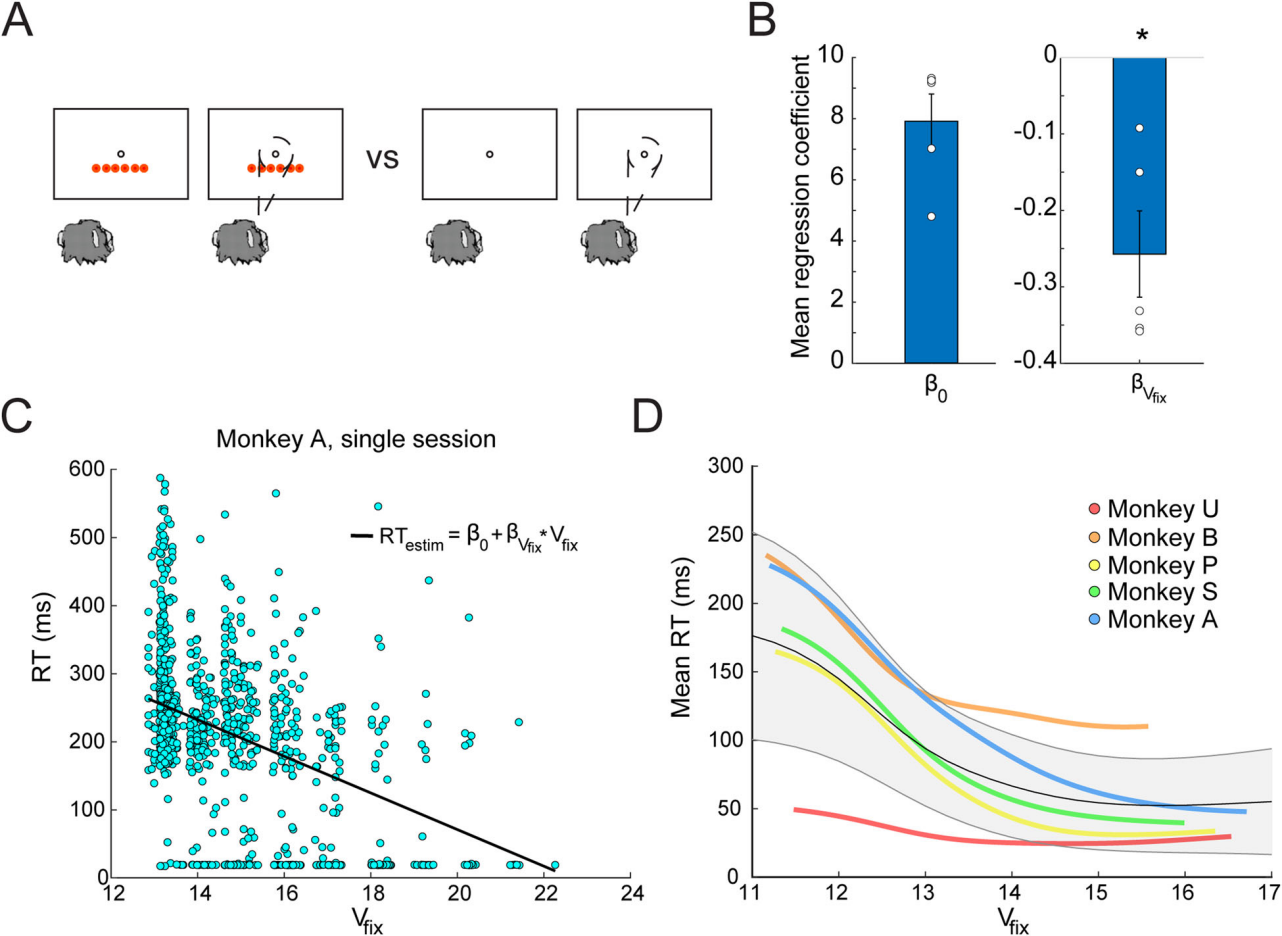
RT to acquire fixation. ***A***, An example case of when motivation might differ in the tokens task at the time of fixation. ***B***, Mean regression coefficients across animals from the linear regression on *V*_fix_ (bar plot) and mean *β* values across sessions for each animal (dots). (* indicates *p* < 0.05.) Note that regressions were conducted on log(RT). ***C***, An example set of RTs from a single session from Monkey A showing a decrease in RT to acquire fixation as *V*_fix_ increases with an overlay of the regression line. Values near zero indicate trials in which the monkey was already within the fixation window when the fixation cue appeared. ***D***, Kernel smoothed, averaged mean RTs for each monkey versus *V*_fix_. Average RTs across sessions are shown for each animal in a different color indicated by the legend. The average of all animals is shown in gray, with error bars indicating the standard deviation across animals.

We next examined whether the MDP state values could be used to predict motivation in monkey behavior. The first question was how the state value might affect the initiation of a trial, which has been previously shown to be affected by motivation (Hamid et al., 2016; Oemisch et al., 2016; Mohebi et al., 2019; Steinmetz et al., 2019). For example, if the monkey has multiple tokens at the start of a trial, might they be more motivated to initiate a trial than in the case when they have no tokens (Fig. 6*A*)? However, token count is not the only task feature that could affect motivation in this task. Thus, we used the value of the fixation state (*V*_fix_) from the MDP to relate all relevant task features (NTk, TSCO, TE, NObs) on a trial-by-trial basis to the time it took the animal to acquire the fixation spot. We conducted a linear regression for each session of data from each animal and calculated the average regression coefficient value for each animal (Fig. 6*B*). Mean regression coefficients for *V*_fix_ (*β_V_*_fix_) were significantly less than zero at the group level (*p* = 0.0312, Wilcoxon signed-rank test). All five of the individual distributions of regression coefficients for each animal were statistically significant (*t*_(20) _= −5.78; *t*_(22) _= −9.48; *t*_(16) _= −11.74; *t*_(19) _= −28.18; *t*_(19) _= −19.37; *p* < 0.0001). Thus, as the value of the fixation state increased, RTs decreased (Fig. 6*C*), and this was true for all five monkeys (Fig. 6*B*,*D*).

### Choice RT is related to the value of the cue state and the change in the state value

**Figure 7.**
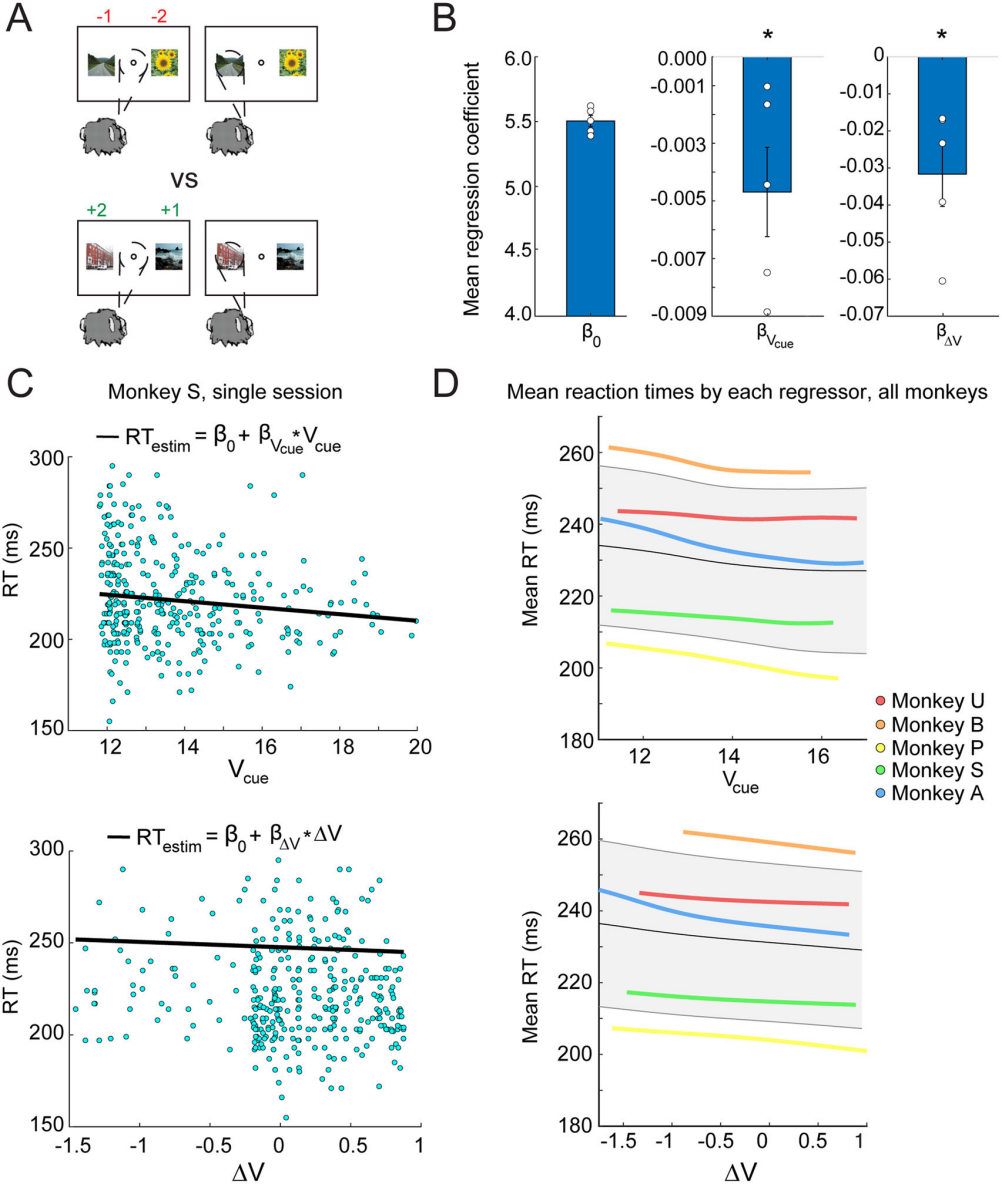
RT to choice. ***A***, An example case of when motivation might differ in the tokens task at the time of image cue presentation. Image credit: Wikimedia Commons (scene images). ***B***, Mean regression coefficients cross animals from the linear regression on *V*_cue_ and Δ*V* (bar plots) and mean *β* values across sessions for each animal (dots). (* indicates *p* < 0.05.) Note that regressions were conducted on log(RT). ***C***, Example set of RTs from a single session from Monkey S showing a decrease in choice RT as *V*_cue_ increases and Δ*V* becomes more positive with regression fits overlayed. ***D***, Kernel smoothed, averaged mean RTs for each monkey versus *V*_cue_ and Δ*V*. Average RTs across sessions are shown for each animal in a different color indicated by the legend. The average of all animals is shown in gray, with error bars indicating the standard deviation across animals.

Next, we asked whether choice RTs were related to the value of the cue state (*V*_cue_) and the change in the value between the cue onset and fixation state (Δ*V* = *V*_cue_-*V*_fix_), which would reflect an impending gain or loss of tokens from selecting a cue. For example, if the cue condition was loss versus loss (−1 vs −2), the monkey might be slower to choose an option than in the case of gain versus gain (+1 vs +2) where there is a preferred option (Fig. 7*A*). We conducted a linear regression of *V*_cue_ and Δ*V* on RTs for each session of data from each animal and calculated the average regression coefficient for each animal (Fig. 7*B*). Mean regression coefficients *β_V_*_cue_ and *β*_Δ_*_V_* were significantly less than zero at the group level (*p* = 0.0312, Wilcoxon signed-rank test). This meant that as the value of the cue state increased, RTs became faster, and as the change in cue value became more positive, RTs also became faster (Fig. 7*C*,*D*). Four of the five individual monkey distributions of *β_V_*_cue_, where session was the repeat, were statistically significant (*t*_(20) _= −2.19; *p* < 0.05; *t*_(22) _= −6.62, *p* < 0.0001; *t*_(16) _= −3.44; *p* < 0.005; *t*_(19) _= −1.98; *p* = 0.06; *t*_(19) _= −14.78; *p* < 0.0001). Five of the five individual distributions of *β*_Δ_*_V_* were statistically significant (*t*_(20) _= −7.29; *p* < 0.0001; *t*_(22) _= −8.86; *p* < 0.0001; *t*_(16) _= −3.22; *p* < 0.01; *t*_(19) _= −3.89; *p* < 0.001; *t*_(19) _= −9.98; *p* < 0.0001). In summary, this demonstrated that as the value of the cue state was higher, choice RTs were faster for all animals. These analyses also demonstrated that when the change in the state value from fixation to cue (Δ*V*) was positive, RTs were also faster.

### The probability of the monkey aborting a trial is related to the value of the cue state

**Figure 8.**
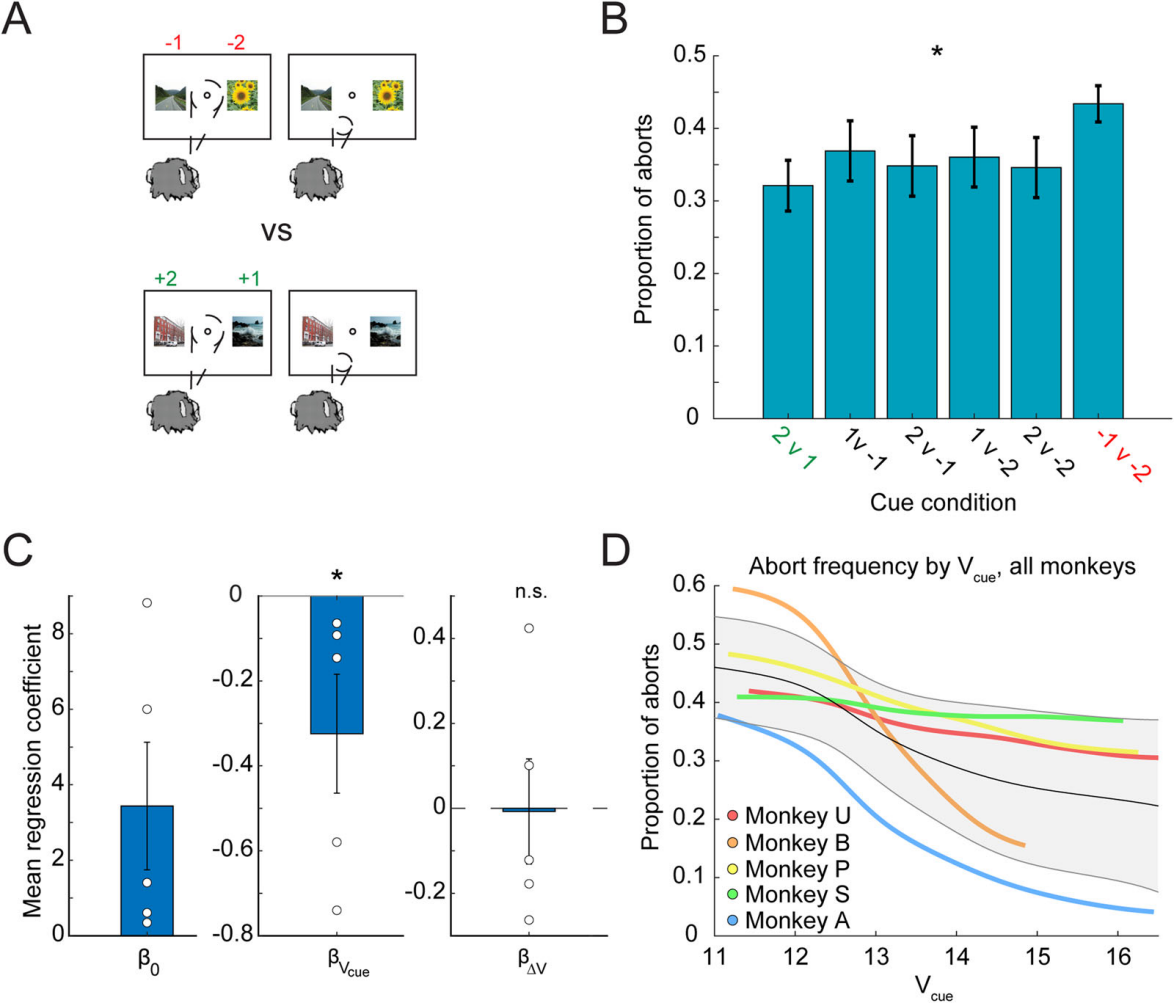
Probability of aborting a trial. ***A***, An example case of when motivation to complete a trial might differ in the tokens task at the time of image cue presentation. Image credit: Wikimedia Commons (scene images). ***B***, Average proportion of aborts in each task condition (SEM average across *n* = 5 monkeys; * indicates *p* < 0.05). ***C***, Mean regression coefficients across animals for the logistic regression on *V*_cue_ and Δ*V*. Only the regressor for *V*_cue_ was statistically significant. ***D***, Kernel smoothed, averaged mean RTs for each monkey versus *V*_cue_. Average proportions of aborts across sessions are shown for each animal in a different color indicated by the legend. The average of all animals is shown in gray, with error bars indicating the standard deviation across animals.

To investigate the relationship between the completion of a trial and the state value, we related the frequency of trial aborts to the state value by looking at all trials in a session and analyzing both complete and incomplete trials. If the monkey moved his eyes outside the fixation window during fixation, did not choose a cue, or did not hold the cue long enough, the trial was aborted and repeated. Given that the monkeys do not learn to pick the smaller loss well in the loss versus loss condition (Fig. 5), it might be more likely that the animal aborts these trials to avoid losing tokens (Fig. 8*A*). Indeed, past work has shown that monkeys are more likely to abort cue conditions with two loss cues (Taswell et al., 2018). We found a significant effect of cue condition on the frequency of aborts (mixed-effect ANOVA, main effect, cue condition *F*_(5,29)_ = 7.86; *p* < 0.001; random effect, monkey *F*_(4,29)_ = 51.14; Fig. 8*B*). We next asked whether the cue state value and changes in the state value were related to the probability of aborting a trial by conducting logistical regression on the cue state value (*V*_cue_) and the change in the value between the cue state and fixation state (Δ*V* = *V*_cue_-*V*_fix_). The distribution of mean regression coefficients *β_V_*_cue_ was significantly less than zero at the group level (*p* = 0.0312, Wilcoxon signed-rank test), whereas *β*_Δ_*_V_* did not emerge as significant (*p* = 0.4062, Wilcoxon signed-rank test), suggesting that changes in the state value were not the main factor related to abort behavior (Fig. 8*C*). Four of the five individual distributions of *β_V_*_cue_ were statistically significant (*t*_(20) _= −3.33; *p* < 0.01; *t*_(22) _= −19.78; *p* < 0.0001; *t*_(16) _= −3.46; *p* < 0.01; *t*_(19) _= −1.94; *p* = 0.068; *t*_(19) _= −14.78; *p* < 0.0001). Overall trends across animals showed that as the value of the cue state increased, the probability of aborting decreased (Fig. 8*D*).

### All MDP state features contribute to state values

We next examined whether the token count was the only driving force for the correlations found between state values and behavior. As was shown, the current token count strongly influenced the state value (Fig. 4). To assess the contribution of each of the features in the MDP to the regression results, we marginalized across each feature, thus removing the effect of variation in that feature on the state value and recomputed the regressions. For example, to marginalize over the token count, state values for each trial were extracted using only the other features (TSCO, TE, NObs) after averaging over the values for all possible values of the token count. The average of these state values was used as the single-trial state value for the regressions.

**Figure 9.**
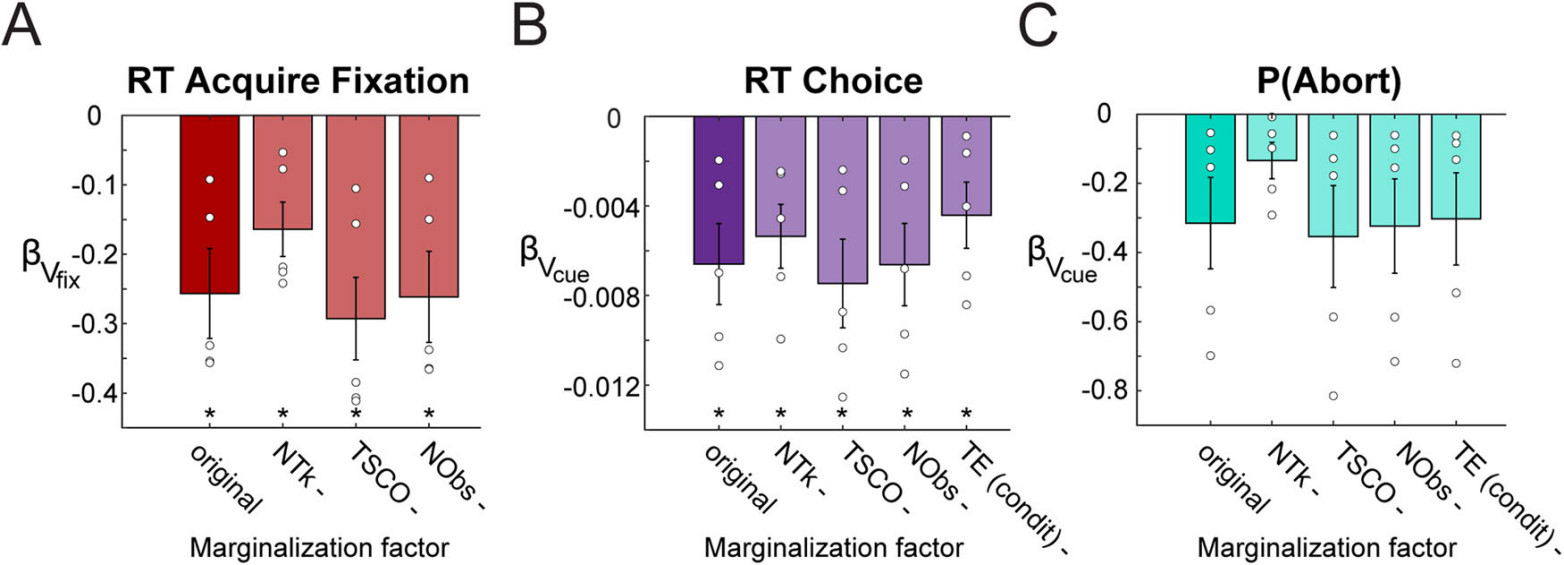
Marginalization over features. Linear regressions for each behavioral feature were recomputed using state values that omitted the effect of a single feature at a time: NTk, TSCO, NObs, TE (condit). Mean regression coefficients across animals are shown (bar plots) and for each subject (dots) for three behavioral features: (***A***) RT to acquire fixation, (***B***) RT to choice, (***C***) probability of aborting trials. Error bars indicate SEM.

For RT to acquire fixation, all distributions of regression coefficients remained statistically significant for each marginalized version of the regression (Fig. 9*A*). Removing the token count from the regression had the largest effect on reducing the relationship between *V*_fix_ and RT to acquire fixation. Removing TSCO and NObs in the regression for RT to acquire led to an increase in *β* values, which suggests these factors interact and affect the regression but are less important than the token count in the regression. For both choice RT and the probability of aborting a trial, regressions were recomputed using only one regressor, for *V*_cue_. This was because removing the cue condition from the regression caused Δ*V* = *V*_cue_-*V*_fix_ to go to zero and therefore made the regressions uninterpretable. Marginalizing over cue condition or tokens in the regression for choice RTs reduced the magnitude of the regression coefficients (Fig. 9*B*). This reflects a weaker relationship between the state value and RTs without these features. In the logistic regression for aborts, marginalizing over tokens also had the largest effect on the regressors, but did not eliminate the relationship between the state value and the probability of aborting a trial (Fig. 9*C*). Taken together, these analyses show that the NTk strongly affects all behavioral measures but is not the only factor leading to the relationships between behavior and the state value.

To further investigate the relative contributions of individual MDP features to the three behaviors, we performed additional multivariate regressions (linear and logistic) with the MDP features and the chosen value derived from the RW model. All coefficient distributions were tested using the same nonparametric Wilcoxon signed-rank test. For RTs to acquire fixation, we conducted a multivariate linear regression on the relevant features NTk, TSCO, and NObs (TE is always the same). The distribution of mean regression coefficients *β*_NTk_ was significantly less than zero at the group level (*p* = 0.0312). For *β*_TSCO_ and *β*_NObs_, these regressors were not statistically significant at the group level (*p* = 0.1562, *p* = 0.3125, respectively).

For choice RTs, two additional regressions were performed. Multivariate regression on the relevant features NTk, TSCO, NObs, and cue condition (as a categorical variable) was performed. The distribution of mean regression coefficients *β*_NTk_ was not significantly different than zero (*p* = 0.2188). However, the distributions of mean coefficients were significantly greater than zero for *β*_TSCO_ and *β*_NObs_ (*p* = 0.0312). Individual regressors for cue conditions were significant for Conditions 3 (2 vs −1), 5 (2 vs −2), and 6 (−2 vs −1; *p* = 0.0312) and not significant for Conditions 2 (1 vs −1) and 4 (1 vs −2), with Condition 1 (2 vs 1) as the reference condition (*p* = 0.0625). For choice RTs, we also performed a linear regression on the chosen value from the RW model, and the mean distribution *β*_RWChosenValue_ was statistically significant at the group level (*p* = 0.0312).

To analyze abort trials, aborts were separated into two categories: those which happened during fixation (i.e., did not acquire, did not hold) and those which happened during choice (i.e., did not choose, did not hold). Without the concept of state, these types of aborts must be analyzed separately as the cue condition is assumed to be unknown when abort happens during fixation, because that occurs before the cue condition onset. Thus, two additional regressions were performed, one on fixation aborts with relevant features that included NTk, TSCO, and NObs and one on choice aborts on NTk, TSCO, NObs, and cue condition as a categorical variable. For fixation aborts, the distribution of mean regression coefficients *β*_NTk_ was significantly different than zero (*p* = 0.0312). *β*_TSCO_ was not significant (*p* = 0.1562), and *β*_NObs_ was significant at the group level (*p* = 0.0312). For choice aborts, the distribution of mean regression coefficients *β*_NTk_ was not significantly different than zero (*p* = 0.0938). *β*_TSCO_ was significant at the group level (*p* = 0.0312), and *β*_NObs_ was not (*p* = 0.0938). Individual regressors for cue conditions were significant for Conditions 2 (1 vs −1), 4 (1 vs −2), 5 (2 vs −2), and 6 (−2 vs −1; *p* = 0.0312) and not significant for Condition 3 (2 vs −1; *p* = 0.0625) with Condition 1 (2 vs 1) as the reference condition. Taken together, these additional analyses demonstrate the utility of a state-based model to integrate across all reward-predictive features in order to relate the evolution of the value with motivation and behavior.

## Discussion

In this study, we aimed to investigate the relationship between symbolic reinforcers and motivation. Monkeys learned to make choices to maximize gains and minimize losses of tokens. The tokens were symbolic reinforcers that represented future juice rewards. We designed an MDP model to capture the relationship between features of the task (integrated into states) that predict the reward in order to compute the state value at subtrial resolution. We then related these state values to three behavioral measures of motivation. The state space for the task included the NTk, TSCO, TE, and NObs. We found that RTs to acquire fixation, choice RTs, and the probability of aborting a trial were significantly related to the state value and changes in the state value (except abort probability). Furthermore, we demonstrated that state values were dependent on all state features, not just the NTk. The number of tokens did, however, often have a large effect. These relationships between the state value and behavior cannot be captured by simpler models such as the RW model, as these models are stateless and therefore cannot capture state values that depend on future rewards, nor can they account separately for tokens versus primary rewards. Given that the MDP also allows for modeling trial state-dependent values, it can also be used in future work to understand the neural circuitry relevant to the task.

Past work has shown that symbolic (or secondary) reinforcers can drive learning and have motivational properties similar to those of primary rewards (Wolfe, 1936; Wyckoff, 1959; Jackson and Hackenberg, 1996; Sousa and Matsuzawa, 2001; Seo and Lee, 2009; Donahue and Lee, 2015; Farashahi et al., 2018; Taswell et al., 2018, 2023; Beran and Parrish, 2021; Yang et al., 2022). This happens through the propagation of reward information from primary rewards to symbolic rewards (tokens, cue images) and actions associated with the symbolic rewards (choosing a cue image). However, the computational and neural mechanism of this transfer remains unclear. In the task used in this study, tokens and cue images both predict rewards, although in different ways. Cues are stochastically linked to tokens on short timescales, whereas tokens are deterministically linked to the juice on longer timescales. Cues, therefore, predict rewards but only through tokens. The cues also change in each block, which requires rapid learning of the cue values, whereas the relationship between tokens and juice is stable and constant over the course of the experiment. The state-based modeling framework presented here provides a conceptual advance over other available RL models in that it accounts for the differential attributes of cues and tokens and implements a computational mechanism by which symbolic reinforcers can acquire the motivating properties of rewards. Furthermore, the model allows for examining how reward-predicting features interact and how they affect behavioral measures of motivation, including the trial initiation time, choice RTs, and trial aborts, through the lens of the state value.

The time to initiate a trial has been studied previously as a measure of motivation (Hamid et al., 2016; Oemisch et al., 2016; Mohebi et al., 2019; Steinmetz et al., 2019). In a task which required rodents to nose poke after a light went on, rodents were faster, interpreted as increased motivation, when the reward rate was higher (Hamid et al., 2016; Mohebi et al., 2019). When we investigated the relationship between the state value and RTs to acquire fixation, we found that a higher state value correlated with faster RTs to acquire fixation. This also means that on the trials immediately after receiving primary juice rewards (during cash-out), when the state value is lowest, the monkeys are, on average, slower to initiate the next trial. Were the primary juice reinforcer doing all of the reinforcement of taking actions in the task, the time to initiate a trial would be faster immediately after cash-out. This is not what we observed. Thus, symbolic reinforcers have assumed the motivational properties of rewards to encourage the choice to begin work. Thus, symbolic reinforcers have assumed the motivational properties of rewards to encourage the choice to begin work.

Past work on choice RTs has also suggested that reward expectation can influence execution of a choice response (Hollerman et al., 1998; Wrase et al., 2007). Our regressions on the cue state value and changes in the state value from fixation suggested that RTs to choose options were affected by other task factors, including the distance to cash-out, the NTk present, and the desirability or value of the cue condition. This fits with past work that has shown that expected outcomes can affect RTs (Hollerman et al., 1998; Shidara et al., 1998). In the tokens task, once the monkeys knew the values of the cue images, the images served as a similar instruction to the possible outcomes as in past studies. Regressions on the cue state value showed that as the cue state value increased, RTs decreased, as the monkeys learned to anticipate gains from certain cue conditions. Correspondingly, in loss versus loss (−2 vs −1) trials, the monkeys slowed their choices.

Aborted trials can happen for many reasons. In our tokens task, however, we observed a systematic increase in abort trials in the condition involving only loss options (−2 vs −1), which led us to investigate how the cue state value and changes in the state value might correlate with this behavior. Past work on trial abort behavior has shown that aborts (or refusals) occur most often in trials furthest from the reward (La Camera and Richmond, 2008; Inaba et al., 2013) and trials that require the most effort (Pasquereau and Turner, 2013, 2015; Varazzani et al., 2015), suggesting that animals are more motivated to complete a trial when the cost of reaching a reward is lower. Our regression results are consistent with these findings, as monkeys were less likely to abort when the cue state value was higher. Furthermore, in this task, a trial is immediately repeated after an abort until the trial is completed. Thus, there is no inherent value in aborting a trial (i.e., no additional information gained), because with standard temporal discounting, aborting decreases the future expected value by delaying the reward. If abort states were modeled explicitly in the MDP, the model would not choose the abort option without additional parameterization. While we could incorporate parameters to tune each model to abort at the rate each monkey does, we do not have a mechanism for validating these choices.

Our analysis showed that monkeys were motivated to work when they had more tokens. However, as our marginalization over dimensions of the state vector showed, state values and our regression results depend on more than the NTk present. In this task, the higher state value, and therefore higher discounted future expected reward, led to faster trial initiation, faster RTs, and fewer aborts. This has implications for understanding the neural responses, as the time leading up to the receipt of the reward, also known as the anticipatory phase (Knutson et al., 2001; Ernst et al., 2004; Rademacher et al., 2017), has signals that capture the expectation of the future reward, which occurs in the consummatory phase (Dillon et al., 2008; Kumar et al., 2014). Understanding the dynamics of anticipation, motivation, and reward in a single framework allows for linking both processes to fluctuations in neural activity in multiple brain areas.

Within the presented framework, symbolic reinforcers have been recast as dimensions that drive the state value. Past work involving choice tasks and the state value has suggested the existence of a ventral circuit for the representation of the state value (Gläscher et al., 2010; Averbeck and Murray, 2020) and state transitions (Belova et al., 2008; Chan et al., 2021; Kalmbach et al., 2022). It has been hypothesized that distinct ventral and dorsal networks define behavioral goals and orchestrate actions to achieve goals, respectively (Everitt et al., 1999; Cardinal et al., 2002; Averbeck and Costa, 2017; Averbeck and Murray, 2020). In choice tasks, the main behavioral goal is to reach high value states. Recent work has shown correlations between fluctuations in dopamine, and the state value (Hamid et al., 2016), and the local control of dopamine in the ventral striatum, is related to motivation (Mohebi et al., 2019). However, the ventral circuit, which includes the orbital frontal cortex, ventral medial prefrontal cortex, ventral striatum, ventral pallidum, and amygdala, is innervated by dopaminergic projections in multiple sites (Haber, 2014), and thus dopamine may differentially affect processing in each of these areas to support RL and motivation (Berke, 2018; Westbrook and Frank, 2018). Furthermore, recent lesion work has shown that lesions of the ventral striatum and amygdala show only subtle deficits on the performance on the tokens task (Taswell et al., 2018, 2023) but larger deficits in reversal learning tasks (Costa et al., 2016) and tasks requiring switches between action-based and stimulus-based strategies (Rothenhoefer et al., 2017).

The question then becomes: How are connections between symbolic reinforcement, rewards, and actions represented in the brain? Symbolic reinforcers such as tokens could be tracked directly across multiple areas, as a global representation of visual object numerosity (Tudusciuc and Nieder, 2009; Ramirez-Cardenas et al., 2016; Viswanathan and Nieder, 2020), but numerosity does not directly have motivational value. However, symbolic reinforcers can take on a range of identities. Furthermore, other states including the abstract completion of intermediate goals can serve as symbolic reinforcers (Janssen et al., 2022). Furthermore, as the capacity to measure more neural signals simultaneously has advanced, there has been growing evidence that task-related signals are represented across many areas (Dotson et al., 2018; Steinmetz et al., 2019; Fine et al., 2023). Therefore, it is unlikely that there would be a localized neural signature of an individual task feature, as most task features will be represented across many areas. Thus, we must consider how symbolic reinforcers might be mapped onto a distributed representation that allows for flexibility in the identity of the reinforcer and design future experiments with this in mind. Here, we have selected four features to integrate: tokens, temporal distance to reward, TE, and cue observations to measure the state value moment by moment in the task. The model therefore generates values for each task state, including fixation, cue presentation, token outcome, and the ITI.

In summary, we developed a computational framework that quantifies the effect of symbolic reinforcers and other reward-predicting features on the state value, which we were able to then relate to behavioral measures of motivation. In this task, RTs to initiate a trial, choice RTs, and the probability of completing a trial were correlated with the state value and changes in the state value. Our results suggest that symbolic reinforcers and rewards can have similar effects on behavior, which allows for predictions about how symbolic reinforcers might be represented in the brain.
